# A novel nomogram for predicting long-term heart-disease specific survival among older female primary breast cancer patients that underwent chemotherapy: A real-world data retrospective cohort study

**DOI:** 10.3389/fpubh.2022.964609

**Published:** 2022-08-24

**Authors:** Chao Huang, Zichuan Ding, Hao Li, Zongke Zhou, Min Yu

**Affiliations:** ^1^Department of Orthopedics, West China Hospital of Sichuan University, Chengdu, China; ^2^Department of Anesthesiology, North-Kuanren General Hospital, Chongqing, China

**Keywords:** breast cancer, heart disease-specific survival, female, chemotherapy, nomogram, risk classification system, SEER

## Abstract

**Background:**

The past decade has witnessed an improvement in survival rates for breast cancer, with significant inroads achieved in diagnosis and treatment approaches. Even though chemotherapy is effective for this patient population, cardiotoxicity remains a major challenge, especially in older people. It has been established that cardiovascular events are a major cause of death in older female primary breast cancer patients that underwent chemotherapy. In the present study, the independent prognostic factors were identified to develop a novel nomogram for predicting long-term heart disease-specific survival (HDSS) and improving patient management.

**Method:**

Older female primary breast cancer patients that underwent chemotherapy from 2010 to 2015 were retrieved from the Surveillance, Epidemiology, and End Results (SEER) database and randomly assigned to a training cohort and a validation cohort at a ratio of 7:3. HDSS was the primary endpoint of this study. Univariate and multivariate Cox regression analyses were conducted on the training cohort to identify independent prognostic factors of HDSS and construct a nomogram to predict the 5- and 8-year HDSS. The performance of the constructed nomogram was evaluated by calibration curve, receiver operating characteristic (ROC) curve, and decision curve analyses. Finally, a risk classification system was constructed to assist in patient management.

**Result:**

A total of 16,340 patients were included in this study. Multivariate Cox regression analysis identified six independent prognostic factors: age, race, tumor stage, marital status, surgery, and radiotherapy. A nomogram based on these six factors yielded excellent performance, with areas under the curve of the ROC for 5- and 8-year HDSS of 0.759 and 0.727 in the training cohort and 0.718 and 0.747 in the validation cohort. Moreover, the established risk classification system could effectively identify patients at low-, middle-, and high- risk of heart disease-associated death and achieve targeted management.

**Conclusion:**

Independent prognostic factors of HDSS in older female primary breast cancer patients that underwent chemotherapy were determined in this study. A novel nomogram for predicting 5- and 8-year HDSS in this patient population was also established and validated to help physicians during clinical decision-making and screen high-risk patients to improve outcomes.

## Introduction

Cancer is the second most common cause of death in the US, behind heart disease (1). According to the latest data released by the American Cancer Society, the expected number of female breast cancer cases will increase by 287,850 in the US in 2022, leading to an estimated 43,250 deaths. Interestingly, it has been reported that since the 1950s, the incidence of breast cancer has increased by 0.5% per year. However, with early detection of breast cancer through screening, increased sensitization, and improved treatments, breast cancer mortality has fallen by 42% over the past 30 years (2). Increasing age and female gender are reportedly significant risk factors for breast cancer. The risk of developing invasive breast cancer in women under 49, 50–59, 60–69, and older than 70 years old has been reported to be 2.1, 2.4, 3.5, and 7%, respectively (2).

Increasing age is a natural driver of cardiovascular morbidity and mortality in the general population; cardiovascular diseases have been documented to be a significant risk factor for mortality in older females with breast cancer. Abdel-Qadir et al. showed that among breast cancer women aged 66 years or older with no cardiovascular disease, the 10-year risk of breast cancer- and cardiovascular disease-associated death were 11.9 and 7.6%, respectively. Interestingly, among patients with pre-existing cardiovascular disease, the risk of death from cardiovascular disease and breast cancer was comparable for the first 5 years. However, the risk of death from cardiovascular disease exceeded breast cancer over time, with a 10-year cumulative mortality rate of 16.9 and 14.6%, respectively (3). Over the years, anthracycline-based chemotherapy has exhibited high efficacy in treating breast cancer. However, it has been shown that cardiotoxicity and heart failure risks increase with cumulative doses of anthracyclines (4, 5). Accordingly, mortality caused by cardiovascular disease in older breast cancer patients that underwent chemotherapy accounts for poor long-term heart disease specific survival (HDSS).

Although risk factors associated with HDSS in breast cancer have been identified, there is currently no universally accepted scoring system to predict long-term HDSS in this subpopulation. Given that different clinical-pathological variables can affect the patient prognosis, a new approach that integrates key prognostic predictors is warranted to help during treatment selection and improve patient quality of life. Nomograms are nowadays widely accepted as a simple multivariate visualization tool for predicting individual patient survival outcomes, especially in oncology (6, 7). Compared with the tumor-node-metastasis staging system, nomograms can more accurately estimate the survival of individual patients by integrating key variables to aid in clinical decision-making and facilitate the development of precision medicine (6). To our knowledge, no nomogram has been documented in the literature for predicting HDSS in this subpopulation. More in-depth analysis of this subpopulation is necessary to identify prognostic factors associated with HDSS and develop scientifically appropriate cardiovascular mortality prevention measures to improve survival outcomes. Therefore, this study aimed to identify independent prognostic factors associated with HDSS in this subpopulation by analyzing relevant data from the Surveillance, Epidemiology, and End Results (SEER) database and to develop a novel nomogram for predicting the 5- and 8-year HDSS.

## Methods

### Database

The SEER database (https://seer.cancer.gov/seerstat/) collects data from 18 separate cancer registries covering ~30% of the US population. It was used in this retrospective cohort study (8). SEER Stat software v8.3.9.2 was used to identify the relevant data of this subpopulation in the SEER database from 2000 to 2018 with the reference number 16336-Nov2020 [Incidence-SEER Research Plus Data, 18 Registries, Nov 2020 Sub (2000–2018)]. Since SEER is a publicly available database, and the acquired data does not include personal information, no ethics approval and informed consent are required. This research was conducted following the Strengthening the Reporting of Observational Studies in Epidemiology (STROBE) guidelines (9).

### Patient selection

The inclusion criteria consisted of (i) patients with the following site-specific codes for cancer that originated in the breast: C50.0 (Nipple), C50.1 (Central portion of the breast), C50.2 (Upper-inner quadrant of the breast), C50.3 (Lower-inner quadrant of the breast), C50.4 (Upper-outer quadrant of the breast), C50.5 (Lower-outer quadrant of the breast), C50.6 (Axillary tail of the breast), C50.8 (Overlapping lesion of the breast), and C50.9 (Breast, NOS) (10); (ii) older female (age ≥65) (11–14); (iii) patient underwent chemotherapy; (iv) “diseases of the heart” and “alive” were used to classify patient death classification according to “COD to site rec KM”; (v) primary tumor; and (6) complete follow-up data available. Patients were excluded for the following reasons: (i) male; (ii) no chemotherapy; (iii) age <65; (iv) breast cancer is not the primary tumor; (v) demographic and clinical data, including age, race, marital status, Breast-Adjusted AJCC 6th Stage (tumor stage), tumor grade, surgery, radiotherapy, and tumor size, were not available; (vi) survival time < 1 month. Finally, 16,340 patients were included in this study and randomly divided into training (*n* = 11,440) and validation cohorts (*n* = 4,900) according to a ratio of 7:3. The former was used to identify HDSS-related independent prognostic factors and establish a prognostic nomogram and risk classification system for this subpopulation. The latter was used to verify the constructed nomogram and risk classification system.

### Variable definitions

Patient demographic characteristics (age, race, and marital status), tumor factors (tumor size, tumor grade, and tumor stage), disease characteristics (primary site, breast subtype, ER status, PR status, HER2 status, and distant (bone, brain, liver, and lung) metastasis, and treatment information (surgery and radiotherapy) were analyzed in this study. The optimal cut-off values for age and tumor size in the training and validation cohorts determined by the X-tile software were 71 and 76 years old and 22 and 36 mm, respectively (Supplementary File 1) (15). Patients were categorized into white, black, and others (American Indian/AK Native, Asian/Pacific Islander) based on race. Marital status was divided into “married” and “single/other”. Surgery and radiotherapy were categorized into “Yes” and “No” groups. Distant (bone, brain, liver, and lung) metastasis was divided into “Present” and “Absent”. Tumor grades were divided into grades I, II, III, and IV, and clinical tumor stages were classified as stages I, II, III, and IV. The breast subtypes were divided into HR-/HER2- (Triple Negative), HR-/HER2+ (HER2 enriched), HR+/HER2- (Luminal A), and HR+/HER2+ (Luminal B). Moreover, the HER2, PR, and ER statuses were divided into “Positive” or “Negative”. The HDSS, defined as the time interval from the date of diagnosis until death due to heart disease, was the primary endpoint of this study.

### Statistical analysis

All data were analyzed using SPSS (version 22.0) and R (version 4.0.3) software. A *p*-value <0.05 was statistically significant. First, values were assigned to each variable included in this study. The statistical difference between the enrolled variables was identified using the Kaplan-Meier method and univariate Cox regression analysis. Then, variables with a *p*-value <0.05 were incorporated into a multivariate Cox regression analysis to eliminate confounding effects and identify HDSS-related independent prognostic factors in this subpopulation. HDSS-related independent prognostic factors were then used to construct a nomogram to predict 5- and 8-year HDSS. The corresponding scores of the independent prognostic factors in the HDSS nomogram were obtained. Then, the bootstrap-corrected concordance index (C-index) and calibration curves were constructed to verify the prediction and discrimination performance of the nomogram, and a decision curve analysis (DCA) was constructed to demonstrate the clinical utility value of the nomogram. The discriminative power of the nomogram was assessed by constructing the receiver operating characteristic (ROC) curves for the 5- and 8-year HDSS based on the area under the curve (AUC) values of the corresponding variables. In addition, the total score was calculated as the sum of the scores corresponding to the HDSS-related independent prognostic factors, and the optimal cut-off value for the total score was obtained using the X-tile software. Then, a risk classification system was established to stratify the cardiovascular mortality risk of this subpopulation into low-, middle-, and high-risk subgroups. Finally, Kaplan-Meier method was used to identify the differences between the three risk subgroups.

## Results

### Demographic and clinicopathologic characteristics

16,340 older female primary breast cancer patients that underwent chemotherapy retrieved from the SEER database were randomly divided into training (*n* = 11,440, 70%) and validation (*n* = 4,900, 30%) cohorts. The majority of patients were aged between 65 and 70 years old (*n* = 10,013, 61.28%), white (*n* = 13,225, 80.94%), and married (*n* = 9,309, 56.97%). No significant difference was found between low-grade (grade I–II) and high-grade (grade III–IV) tumors. Moreover, low-stage (stage I–II) tumors occupied a higher proportion (78.34%) of cases than high-stage (stage III–IV) tumors. The size of most tumors was <22 mm, while C50.4, C50.8, and C50.2 represented the top three primary sites, accounting for 69.38%. The incidence of distant metastases was relatively low. Besides, luminal A was the most common molecular subtype, accounting for 51.51%. Most patients were classified as ER-positive (*n* = 11,393, 69.72%), PR-positive (*n* = 9,085, 55.60%), and HER2-negative (*n* = 11,643, 71.25%). As for the treatment, 97.3 and 62.93% of patients underwent surgery and radiotherapy, respectively (Table 1).

**Table 1:** The baseline demographic and clinicopathologic characteristics of the HDSS-related variables of older female primary breast cancer patients that underwent chemotherapy.

**Variables**	**Training cohort**		**Validation cohort**		**Total**	
	**11,440**	**70.00%**	**4,900**	**30.00%**	**16,340**	**100.00%**
**Age (years)**						
65–70	6,953	60.78%	3,060	62.45%	10,013	61.28%
71–76	3,231	28.24%	1,361	27.78%	4,592	28.10%
>76	1,256	10.98%	479	9.77%	1,735	10.62%
**Race**						
Black	1,312	11.47%	542	11.06%	1,854	11.35%
White	9,246	80.82%	3,979	81.20%	13,225	80.94%
Other	882	7.71%	379	7.74%	1261	7.71%
**Marital status**						
Single/other	4,980	43.53%	2,051	41.86%	7,031	43.03%
Married	6,460	56.47%	2,849	58.14%	9,309	56.97%
**Primary site**						
C50.0 (Nipple)	54	0.47%	12	0.24%	66	0.40%
C50.1 (Central portion of breast)	665	5.81%	273	5.57%	938	5.74%
C50.2 (Upper-inner quadrant of breast)	1,264	11.05%	548	11.18%	1,812	11.09%
C50.3 (Lower-inner quadrant of breast)	622	5.44%	289	5.90%	911	5.58%
C50.4 (Upper-outer quadrant of breast)	3,999	34.96%	1,707	34.84%	5,706	34.92%
C50.5 (Lower-outer quadrant of breast)	940	8.22%	391	7.98%	1,331	8.15%
C50.6 (Axillary tail of breast)	58	0.51%	18	0.37%	76	0.47%
C50.8 (Overlapping lesion of breast)	2,674	23.37%	1,144	23.35%	3,818	23.37%
C50.9 (Breast, NOS)	1,164	10.17%	518	10.57%	1,682	10.29%
**Tumor grade**						
I	996	8.70%	448	9.14%	1,444	8.84%
II	4,666	40.79%	1,949	39.78%	6,615	40.48%
III	5,744	50.21%	2,485	50.71%	8,229	50.36%
IV	34	0.30%	18	0.37%	52	0.32%
**Tumor stage**						
I	3,250	28.40%	1,490	30.40%	4,740	29.01%
II	5,715	49.96%	2,345	47.86%	8,060	49.33%
III	2,220	19.41%	968	19.76%	3,188	19.51%
IV	255	2.23%	97	1.98%	352	2.15%
**Tumor size (mm)**						
<22	5,466	47.78%	2,448	49.96%	7,914	48.44%
22-36	3,676	32.13%	1,480	30.20%	5,156	31.55%
>36	2,298	20.09%	972	19.84%	3,270	20.01%
**Breast subtype**						
HR-/HER2- (Triple Negative)	2,205	19.27%	1,022	20.86%	3,227	19.75%
HR-/HER2+ (HER2 enriched)	1,060	9.27%	393	8.02%	1,453	8.89%
HR+/HER2- (Luminal A)	5,903	51.60%	2,513	51.28%	8,416	51.51%
HR+/HER2+ (Luminal B)	2,272	19.86%	972	19.84%	3,244	19.85%
**ER status**						
Negative	3,449	30.15%	1,498	30.57%	4,947	30.28%
Positive	7,991	69.85%	3,402	69.43%	11,393	69.72%
**PR status**						
Negative	5,096	44.55%	2,159	44.06%	7,255	44.40%
Positive	6,344	55.45%	2,741	55.94%	9,085	55.60%
**HER2 status**						
Negative	8,108	70.87%	3,535	72.14%	11,643	71.25%
Positive	3,332	29.13%	1,365	27.86%	4,697	28.75%
**Radiotherapy**						
No	4,275	37.37%	1,783	36.39%	6,058	37.07%
Yes	7,165	62.63%	3,117	63.61%	10,282	62.93%
**Surgery**						
No	335	2.93%	106	2.16%	441	2.70%
Yes	11,105	97.07%	4,794	97.84%	15,899	97.30%
**Bone metastasis**						
Absent	11,276	98.57%	4,844	98.86%	16,120	98.65%
Present	164	1.43%	56	1.14%	220	1.35%
**Lung metastasis**						
Absent	11,346	99.18%	4,863	99.24%	16,209	99.20%
Present	94	0.82%	37	0.76%	131	0.80%
**Liver metastasis**						
Absent	11,396	99.62%	4,880	99.59%	16,276	99.61%
Present	44	0.38%	20	0.41%	64	0.39%
**Brain metastasis**						
Absent	11,434	99.95%	4,897	99.94%	16,331	99.94%
Present	6	0.05%	3	0.06%	9	0.06%

HDSS, heart disease-specific survival.

### Identification of independent prognostic factors for HDSS

**Figure 1:**
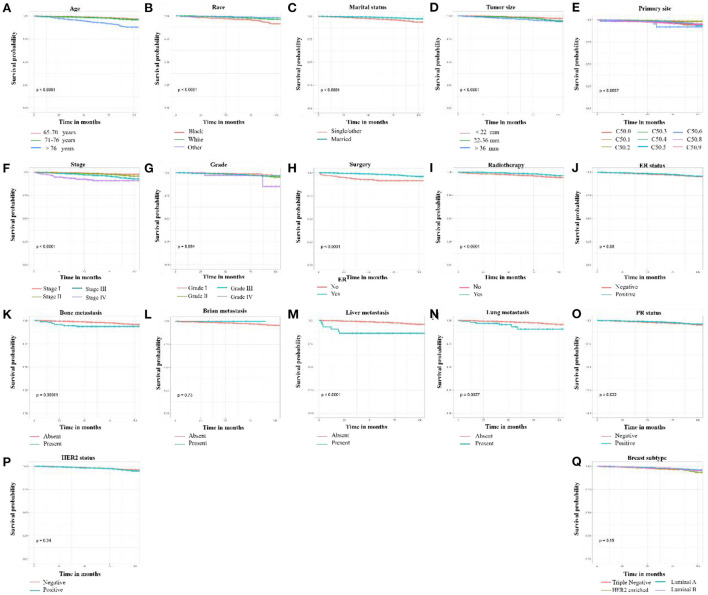
Kaplan-Meier curves of HDSS-related variables in older female primary breast cancer patients that underwent chemotherapy. **(A)** age, **(B)** race, **(C)** marital status, **(D)** tumor size, **(E)** primary site, **(F)** tumor stage, **(G)** tumor grade, **(H)** surgery, **(I)** radiotherapy, **(J)** ER status, **(K)** bone metastasis, **(L)** brain metastasis, **(M)** liver metastasis, **(N)** lung metastasis, **(O)** PR status, **(P)** HER2 status, and **(Q)** breast subtype.

According to the results of univariate Cox regression analysis and Kaplan–Meier curves, age, race, marital status, primary site, tumor grade, tumor stage, tumor size, breast subtype, PR status, surgery, radiotherapy, and distant (bone, liver, and lung) metastasis were significantly associated with HDSS (*p* < 0.05). In contrast, no significant difference in ER status, HER2 status, and brain metastasis were found (Figure 1). Then, HDSS-related variables with a *p*-value<0.05 during univariate Cox regression analysis were used to perform multivariate Cox regression analysis to eliminate the effects of confounding variables. The results showed that age, race, marital status, tumor stage, surgery, and radiotherapy were independent prognostic factors of HDSS in this subpopulation (Table 2).

**Table 2:** The univariate and multivariate Cox regression analyses of the HDSS-related variables of older female primary breast cancer patients that underwent chemotherapy.

**Variables**	**Univariate analysis**		**Multivariate analysis**	
	**OR (95% CI)**	***p* value**	**OR (95% CI)**	***p* value**
**Age (years)**				
65–70	Reference		Reference	
71–76	1.343 (1.000–1.804)	0.050	1.260 (0.937–1.695)	0.125
>76	4.727 (3.598–6.210)	≤0.001	3.861 (2.927–5.093)	≤0.001
**Race**				
Black	Reference		Reference	
White	0.469 (0.353–0.623)	≤0.001	0.542 (0.406–0.724)	≤0.001
Other	0.291 (0.157–0.541)	≤0.001	0.337 (0.181–0.627)	≤0.001
**Marital status**				
Single/other	Reference		Reference	
Married	0.423 (0.331–0.540)	≤0.001	0.544 (0.423–0.698)	≤0.001
**Primary site**				
C50.0 (Nipple)	Reference		Reference	
C50.1 (Central portion of breast)	1.684 (0.227–12.522)	0.610	1.922 (0.258–14.297)	0.523
C50.2 (Upper-inner quadrant of breast)	0.446 (0.058–3.454)	0.439	0.586 (0.076–4.542)	0.609
C50.3 (Lower-inner quadrant of breast)	1.468 (0.196–10.999)	0.709	1.989 (0.265–14.916)	0.503
C50.4 (Upper-outer quadrant of breast)	1.224 (0.171–8.778)	0.841	1.623 (0.226–11.645)	0.630
C50.5 (Lower-outer quadrant of breast)	1.208 (0.163–8.960)	0.854	1.660 (0.224–12.325)	0.620
C50.6 (Axillary tail of breast)	2.736 (0.285–26.306)	0.383	2.950 (0.306–28.416)	0.349
C50.8 (Overlapping lesion of breast)	1.328 (0.184–9.568)	0.778	1.800 (0.250–12.972)	0.560
C50.9 (Breast, NOS)	1.737 (0.239–12.638)	0.585	1.909 (0.262–13.893)	0.523
**Tumor grade**				
I	Reference			
II	1.649 (0.979–2.778)	0.060		
III	1.571 (0.937–2.635)	0.087		
IV	4.487 (1.307–15.404)	0.017		
**Tumor stage**				
I	Reference		Reference	
II	1.521 (1.085–2.133)	0.015	1.354 (0.964–1.901)	0.081
III	2.796 (1.959–3.992)	≤0.001	2.438 (1.697–3.503)	≤0.001
IV	6.965 (4.118–11.779)	≤0.001	3.045 (1.620–5.722)	≤0.001
**Tumor size (mm)**				
<22	Reference			
22–36	1.631 (1.226–2.169)	≤0.001		
>36	2.449 (1.832–3.275)	≤0.001		
**Breast subtype**				
HR–/HER2– (Triple Negative)	Reference			
HR–/HER2+ (HER2 enriched)	1.063 (0.695–1.627)	0.778		
HR+/HER2– (Luminal A)	0.752 (0.557–1.016)	0.063		
HR+/HER2+ (Luminal B)	0.859 (0.597–1.236)	0.413		
**ER status**				
Negative	Reference			
Positive	0.803 (0.628–1.027)	0.081		
**PR status**				
Negative	Reference			
Positive	0.762 (0.603–0.963)	0.023		
**HER2 status**				
Negative	Reference			
Positive	1.131 (0.877–1.458)	0.343		
**Radiotherapy**				
No	Reference		Reference	
Yes	0.465 (0.367–0.588)	≤0.001	0.513 (0.401–0.656)	≤0.001
**Surgery**				
No	Reference		Reference	
Yes	0.222 (0.148–0.333)	≤0.001	0.552 (0.333–0.914)	0.021
**Bone metastasis**				
Absent	Reference			
Present	3.256 (1.731–6.125)	≤0.001		
**Lung metastasis**				
Absent	Reference			
Present	3.218 (1.433–7.227)	0.005		
**Liver metastasis**				
Absent	Reference			
Present	7.590 (3.379–17.052)	≤0.001		
**Brain metastasis**				
Absent	Reference			
Present	0.050 (0.000-4323712331)	0.815		

HDSS: heart disease-specific survival.

### Establishment and verification of the prognostic nomogram for HDSS

**Figure 2:**
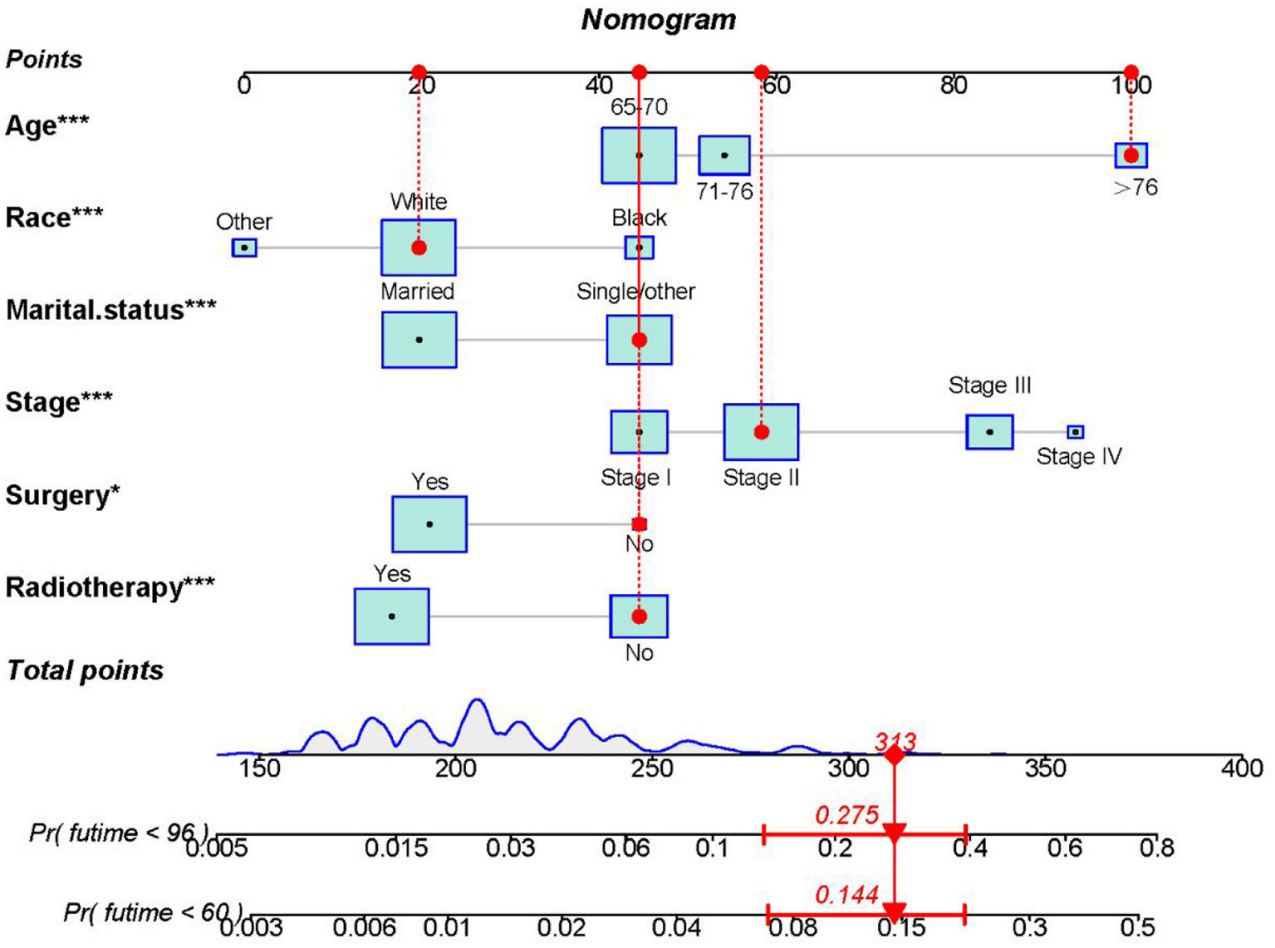
The nomogram was constructed to predict the 5- and 8-year HDSS in older female primary breast cancer patients that underwent chemotherapy. To calculate the HDSS of an individual patient, point values for each prognostic predictor were obtained by drawing a straight line to the top point row. Next, the corresponding point values were summed to get the total score below. The 5- and 8-year HDSS were obtained by drawing vertical lines from the total score row to the bottom timeline. For example, for an 80-year-old unmarried white race female patient with stage II disease that did not undergo surgery or radiotherapy, the total score is 100 (80 years old) +20 (white race) +45 (single/other) +58 (stage II) +45 (no surgery) +45 (no radiotherapy) = 313, and the corresponding risk of heart disease-associated death at 5- and 8-year are 0.144 and 0.275, while the corresponding HDSS of the patient at 5- and 8-year are 0.856 and 0.725.

**Figure 3:**
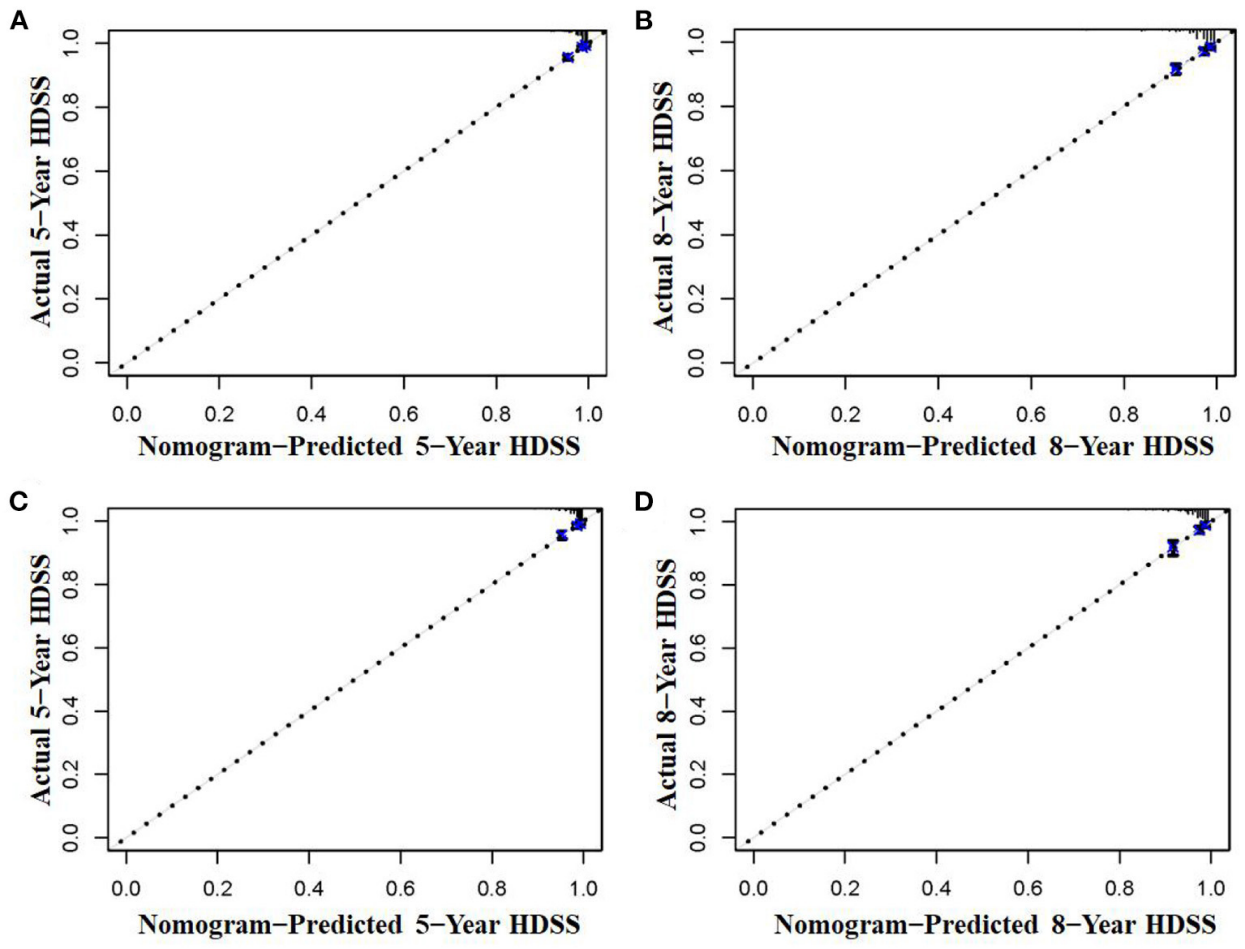
The calibration curves of the nomogram were used to predict the 5- and 8-year HDSS in older female primary breast cancer patients that underwent chemotherapy in the training **(A,B)** and validation cohorts **(C,D)**.

**Figure 4:**
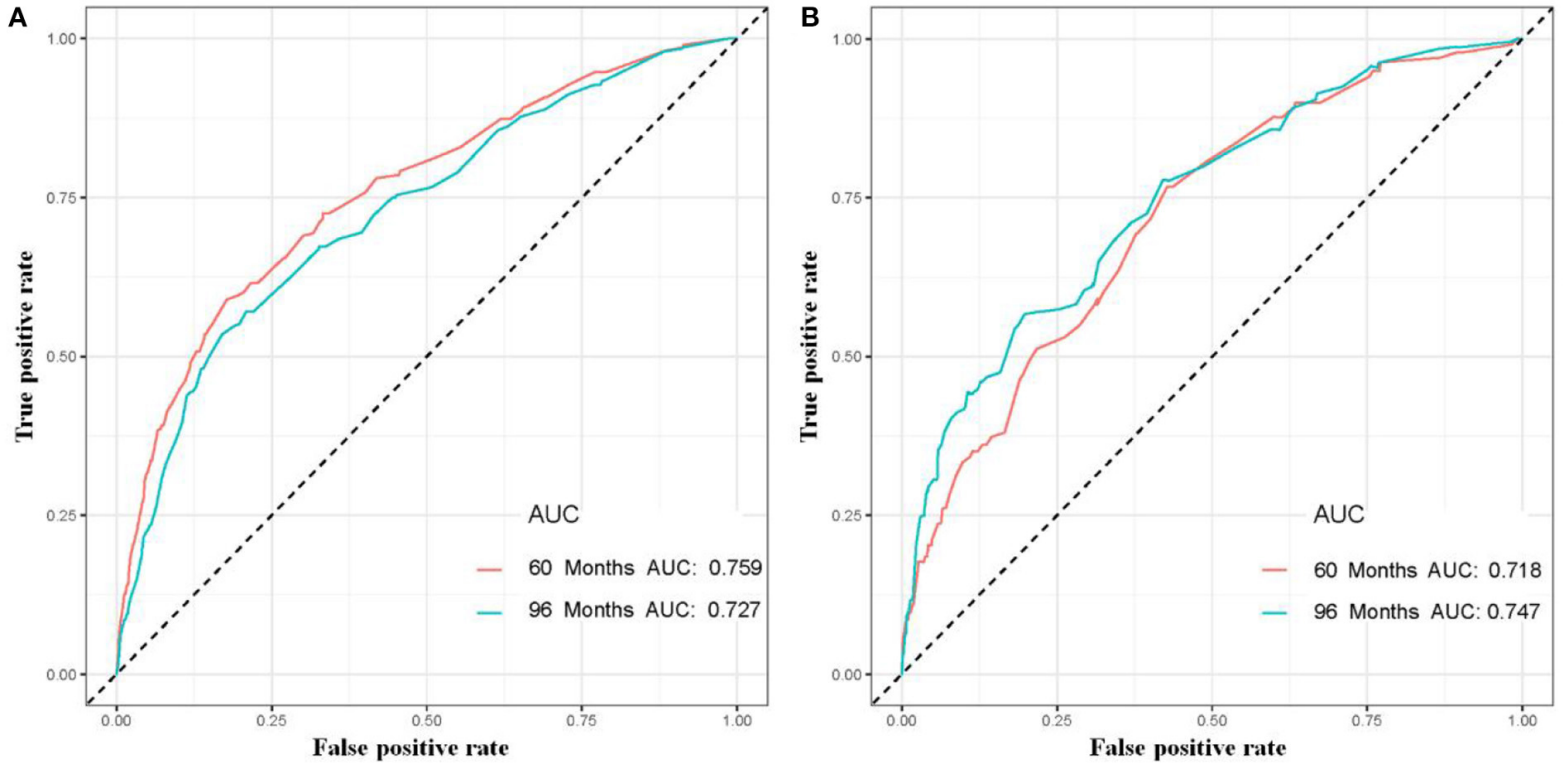
The 5- and 8-year receiver operating characteristic curves of older female primary breast cancer patients that underwent chemotherapy in the training **(A)** and validation **(B)** cohorts.

**Figure 5:**
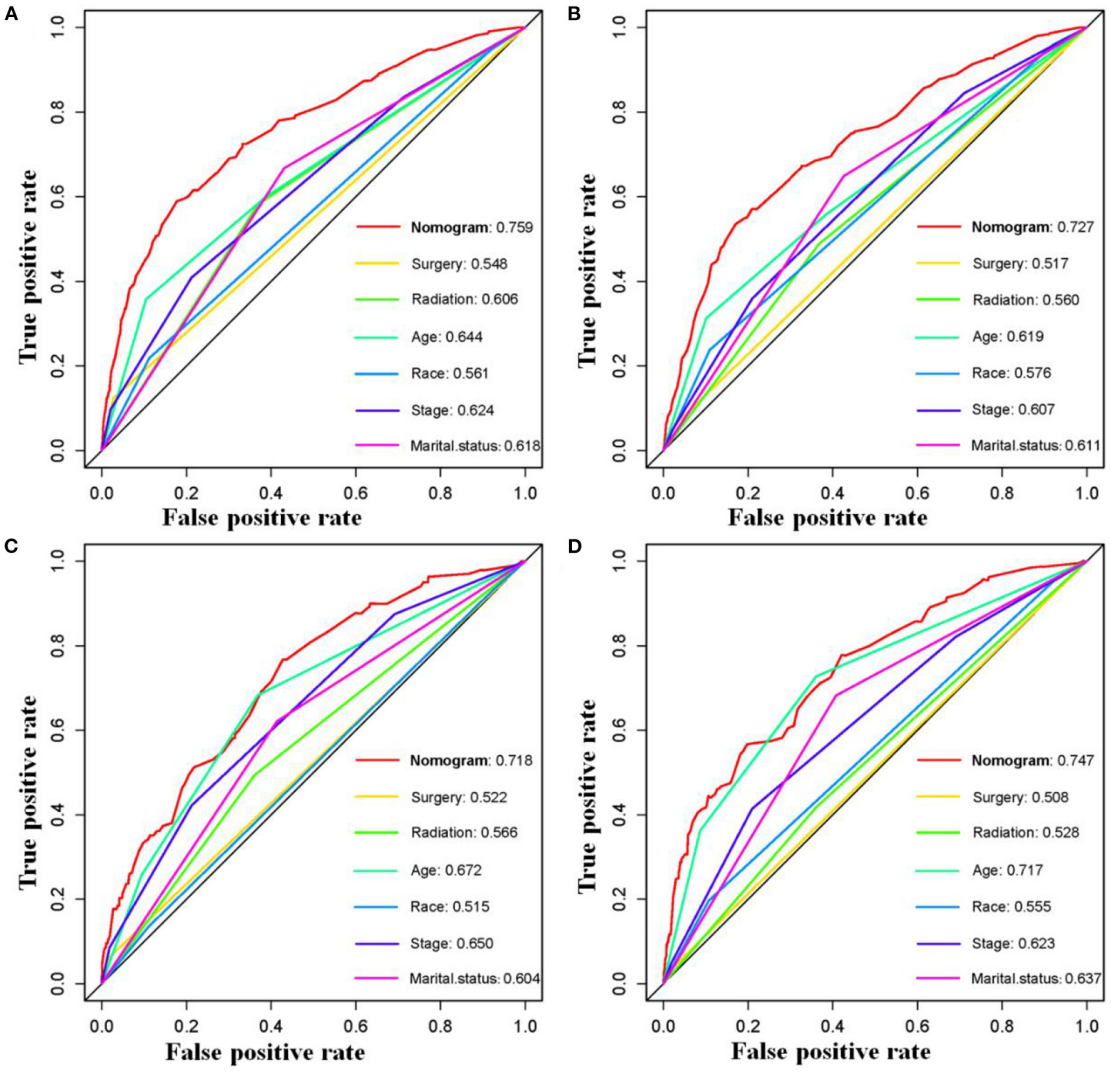
Comparison of prediction accuracy between the constructed novel nomogram and each HDSS-related independent prognostic factors in older female primary breast cancer patients that underwent chemotherapy at 5-**(A)** and 8-**(B)** year in the training cohort and 5-**(C)** and 8-**(D)** year in the validation cohort, respectively.

**Figure 6:**
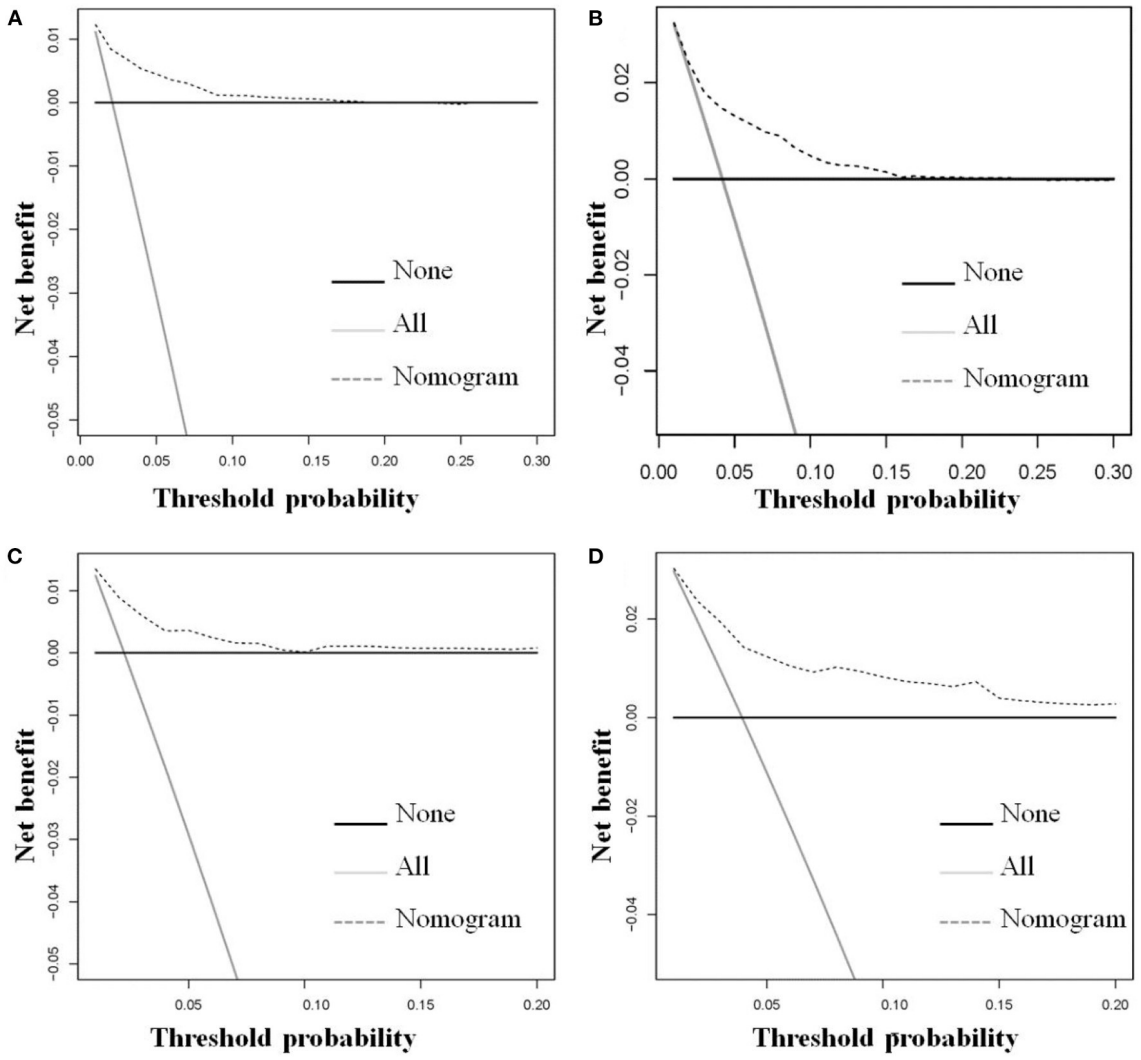
The decision curve analysis of the constructed novel nomogram was used to predict the 5-**(A)** and 8-**(B)** year HDSS in the training cohort and the 5-**(C)** and 8-**(D)** year HDSS in the validation cohort for older female primary breast cancer patients that underwent chemotherapy.

The six aforementioned HDSS-related independent prognostic factors were used to establish a prognostic nomogram for predicting long-term HDSS in older female primary breast cancer patients that underwent chemotherapy (Figure 2). As shown in Figure 2, the corresponding point value of the independent prognostic factors in the HDSS nomogram were obtained by drawing a straight line to the top point row and then were summed to get the total point. The 5- and 8-year HDSS were obtained by drawing vertical lines from the total point row to the bottom timeline. A good prognosis was found for 65–70 years old married patients of other races (American Indian/AK Native, Asian/Pacific Islander) and lower tumor stage (stage I) that underwent surgery and radiotherapy. The calibration curves for 5- and 8-year survival showed good agreement between actual and predicted outcomes based on the constructed nomogram in this subpopulation (Figure 3). The bootstrap-corrected C-index was 0.757 (95% CI: 0.694–0.820) and 0.730 (95% CI: 0.634–0.826) in the training cohort and validation cohort. The AUCs for the 5-year HDSS in the training and validation cohorts were 0.759 and 0.718, respectively. Consistently, the AUCs for the 8-year HDSS in the training and validation cohorts were 0.718 and 0.747, respectively (Figure 4). These findings suggested that the constructed nomogram had good discriminatory power (Figure 4). Moreover, we compared the predictive accuracy between individual independent prognostic factors and the constructed nomogram (Figure 5). The results showed that the AUC of the constructed nomogram was higher than each factor at 5- and 8-years in the training and validation cohorts, indicating that the nomogram yielded a more accurate predictive performance for HDSS in this subpopulation. In addition, DCA showed that the constructed nomogram had high prospects for clinical application (Figure 6).

### Risk classification system for HDSS

**Figure 7:**
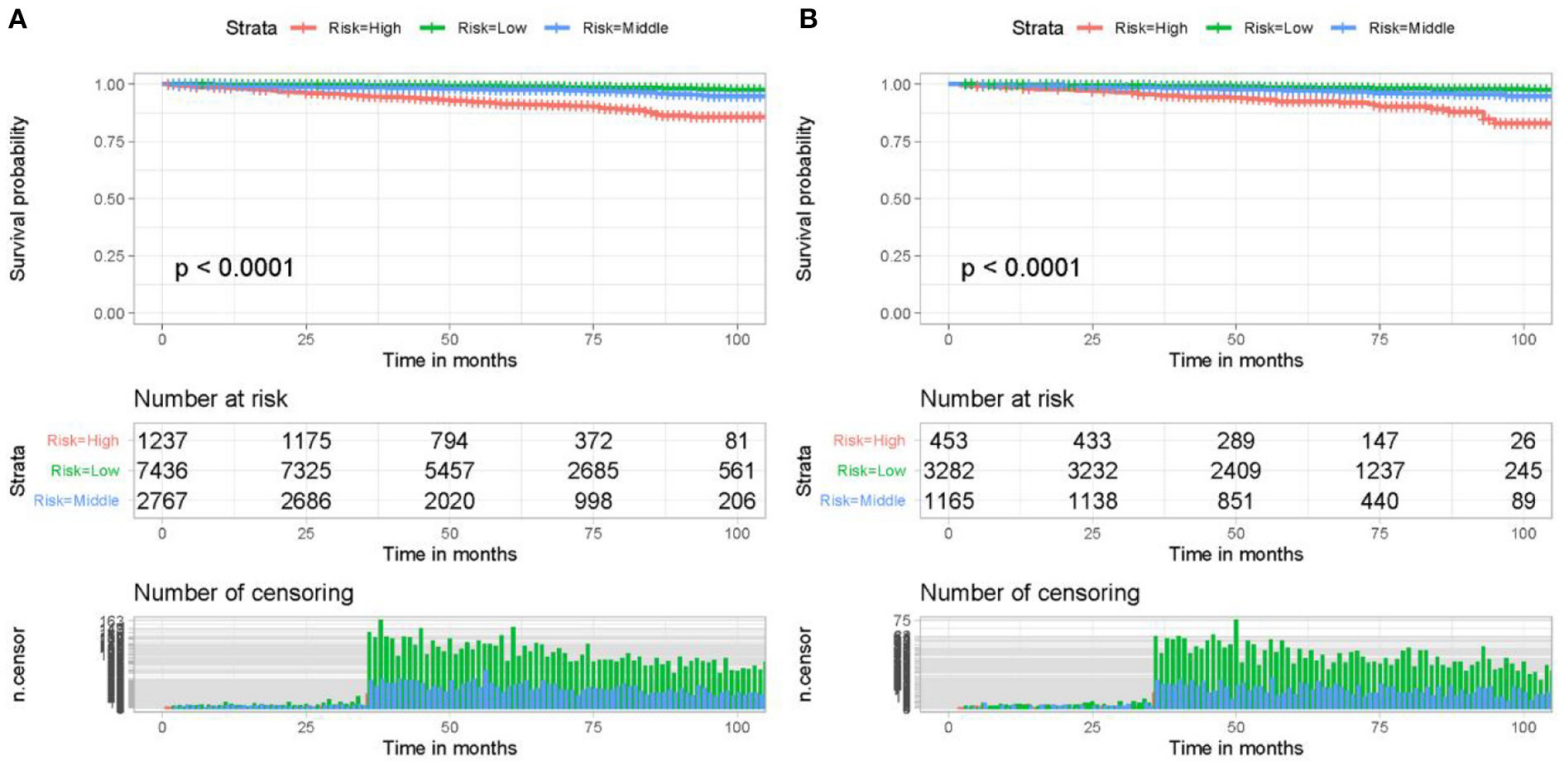
The risk classification system could effectively classify older female primary breast cancer patients that underwent chemotherapy in the training cohort **(A)** and validation cohort **(B)** into three risk subgroups with significant differences. Kaplan-Meier survival curves demonstrated that older female primary breast cancer patients that underwent chemotherapy in the high-risk subgroup had a worse prognosis than those in the low-risk subgroup.

In addition to predicting patient HDSS, it is essential to classify patients based on their cardiovascular mortality risk for individualized management. A cardiovascular mortality risk classification system was constructed using the six HDSS-related independent prognostic factors. Specifically, the total points of all patients were obtained by summing the assigned point values for each independent prognostic factor. The optimal cut-off values for the total point were 223 and 260, according to the results of the X-tile software (Supplementary File 1). Accordingly, patients were further divided into three cardiovascular mortality risk subgroups: low- (<223), middle- (223–260), and high- (>260), and a Kaplan-Meier survival curve was generated (Figure 7). As shown in Figure 7, the risk classification system could effectively classify older female primary breast cancer patients that underwent chemotherapy into three subgroups, indicating that the HDSS nomogram could classify patients based on the cardiovascular mortality risk to improve patient management.

## Discussion

Depending on the tumor stage, subtype, and gene expression results, treatment modalities for breast cancer mainly involve local therapy, including surgery and radiotherapy, and systemic therapy, encompassing chemotherapy, hormone therapy, targeted therapy, and immunotherapy. Among these, chemotherapy is well-established as an effective treatment for breast cancer. Anthracycline-based treatment regimens have been used to treat breast cancer since the 1970s. Nonetheless, its use can reportedly cause cardiac side effects, including cardiomyopathy, ischemia, arrhythmias, and myocardial necrosis, resulting in severe and irreversible left ventricular dysfunction (16, 17). Two main mechanisms can explain this cardiotoxicity: (i) anthracyclines cause myocyte DNA damage, bind to topoisomerase IIβ and disrupt replication (18, 19); (ii) anthracyclines form complexes with intracellular iron, which in turn generate reactive oxygen species that damage DNA, proteins, and lipids, including mitochondrial membranes, and accelerate myocyte death (20, 21). In this regard, Howard et al. showed that doxorubicin-based adjuvant chemotherapy for breast cancer could cause arrhythmias and conduction abnormalities in 2.6% of patients compared to 1% of patients who did not receive doxorubicin (4). Consistently, Guglin et al. showed that anthracyclines could cause atrial fibrillation in 2–10% of patients during or after chemotherapy (22). In addition, cardiotoxicity caused by chemotherapeutic drugs is usually progressive and irreversible. Cardinale et al. showed that recovery of left ventricular function and reduced cardiac events was feasible with early detection and prompt treatment. However, complete left ventricular ejection fraction (LVEF) recovery was not observed in patients treated with chemotherapy over 6 months. On average, LVEF decreases moderately but consistently by ~4% after 3 years of anthracycline exposure (23, 24). Based on these findings, McGowan et al. hypothesized that in the new era of targeted therapy, most breast cancer patients treated with anthracyclines might become the heart disease patients of tomorrow (18).

In addition to anthracycline-based chemotherapy, age is another major risk factor for heart disease. Interestingly, Jeon et al. showed that patients aged ≥50 years old sustained a significant increase in the risk of heart disease compared with those aged <50 years old (16). The incidence of breast cancer increases with age, doubling approximately every 10 years until menopause, where breast cancer growth slows (25). The incidence of heart disease increases steadily with age, but the rate of increase becomes steeper at menopause (26). Gernaat et al. showed that heart disease-related mortality in breast cancer patients ranged from 1.6 to 10.4% (27). In addition, older patients are widely thought to have a poorer prognosis, associated with reduced physical function, cognitive impairment, and comorbidities, such as hypertension, hyperlipidemia, and diabetes. In such circumstances, aggressive treatment is not indicated, and the course of treatment may be shortened, thus affecting the treatment outcome (28, 29). Therefore, there is an urgent need for research on survival and risk factors associated with HDSS in this subpopulation.

In this study, a large-scale population-based data analysis was conducted on 16,340 older female primary breast cancer patients that underwent chemotherapy from the SEER database. Age, race, marital status, tumor stage, surgery, and radiotherapy were identified as independent prognostic factors of HDSS and used to establish a nomogram to predict the HDSS at 5 and 8 years in this subpopulation. The nomogram constructed could provide a quantitative method for HDSS prediction for individual patients in this subpopulation. Importantly, we also used this nomogram to develop a cardiovascular mortality risk classification system that could classify these patients into three risk subgroups: high, middle, and low, allowing clinicians to assess various parameters more objectively and accurately, leading to better patient management.

Herein, we found that race was an independent prognostic factors of HDSS in this subpopulation. Our study showed that black women had a poorer prognosis than white women. Consistently, Berkman et al. showed that among women diagnosed with breast cancer between 1990 and 2010, the heart disease-associated mortality in black women was 6.43 times higher than white women, which may be explained by a lack of regular screening and poor access to health care resources and surgical treatment than whites (30–33). Besides, a shortage of educational resources could contribute to the lack of early recognition and intervention of risk factors associated with cardiovascular disease. Last but not least, lack of exercise, smoking, and shortage of healthy food have been documented to contribute to racial disparities in cardiovascular mortality (33, 34). Indeed, surgery remains the mainstay of breast cancer treatment, allowing effective tumor resection and improving survival. An increasing body of evidence suggests that older female patients with stage IV breast cancer who undergo surgery have better overall survival and cancer-specific survival than those who do not, even in patients with bone metastases (35–38). The similar conclusions were reached in our study, where patients who underwent surgery had significantly higher HDSS than those who did not. In addition, radiotherapy was also a protective factor for HDSS in this subpopulation. Radiotherapy can reduce the tumor size and allow control of distant metastases, reducing the burden of the primary tumor on the body and improving the body's ability to cope with the risk of heart disease.

Interestingly, our study showed that marital status was an independent prognostic factor for HDSS in this patient population. The 5-and 8-year HDSS of married patients was higher than that of divorced, widowed, and single patients regardless of age, race, and tumor grade. Previous studies have shown that married patients, who receive help and encouragement from their spouses, exhibit better compliance with prescribed treatment regimens, and married patients with greater financial resources are more likely to have access to early screening facilities and medical assistance (39–41). Moreover, we observed that the HDSS of patients with stage III/IV disease was lower than those with stage I/II, providing compelling evidence of the importance of improving early diagnosis rates.

Although this study constructed a novel nomogram with good performance for predicting HDSS, some limitations were present. Given the retrospective nature of clinical studies, selection bias was inevitable in our study. Moreover, much uncertainty surrounded the specific cardiovascular causes of death due to the coding system used in the SEER database. Besides, there were missing records for treatment data, such as patient chemotherapy regimen and duration and the presence of coexisting cardiovascular disease at diagnosis. Indeed, further studies in other centers or databases are essential to validate our nomogram.

## Conclusions

Extra caution should be taken by clinicians when treating older female primary breast cancer patients with chemotherapy, given the risk of cardiac disease. Our study showed that unmarried patients with old age, black race, and higher tumor stage with no surgery or radiotherapy had a poor HDSS. Management of heart disease in this patient population should be strengthened, and prompt interventions should be taken to improve outcomes. Our established nomogram and risk classification system for predicting the HDSS at 5 and 8 years could assist physicians in clinical decision-making and managing this subpopulation.

## Data availability statement

The original contributions presented in the study are included in the article/Supplementary material, further inquiries can be directed to the corresponding authors.

## Ethics statement

Ethical review and approval was not required for the study on human participants in accordance with the local legislation and institutional requirements. Written informed consent from the participants' legal guardian/next of kin was not required to participate in this study in accordance with the national legislation and the institutional requirements.

## Author contributions

MY designed and supervised the study. CH and ZD undertook the study, performed the literature review, extracted the data, and analyzed the pooled data. ZD and HL drew the figures and organized the tables. MY and ZZ provided critical comments and revised the manuscript. All authors read and approved the final manuscript.

## Funding

This research was funded by the Regional Innovation and Cooperation Program of Science and Technology Department of Sichuan Province (Grant Number: 2021YFQ0028), and the 1·3·5 Project for Disciplines of Excellence, West China Hospital, Sichuan University (Grant Number: ZYJC18039).

## Conflict of interest

The authors declare that the research was conducted in the absence of any commercial or financial relationships that could be construed as a potential conflict of interest.

## Publisher's note

All claims expressed in this article are solely those of the authors and do not necessarily represent those of their affiliated organizations, or those of the publisher, the editors and the reviewers. Any product that may be evaluated in this article, or claim that may be made by its manufacturer, is not guaranteed or endorsed by the publisher.

## Abbreviations

HDSS: heart disease-specific survival
SEER, Surveillance: Epidemiology and End Results
STROBE: Strengthening the Reporting of Observational Studies in Epidemiology
ROC: receiver operating characteristic
DCA: decision curve analysis
AUC: area under the curve
LVEF: left ventricular ejection fraction.
