# Supplementary material for: A novel nomogram for predicting long-term heart-disease specific survival among older female primary breast cancer patients that underwent chemotherapy: A real-world data retrospective cohort study
Source: Front Public Health. 2022 Aug 24;10:964609. doi: 10.3389/fpubh.2022.964609 (PMC9449644; doi:10.3389/fpubh.2022.964609)
Supplement: Supplementary file 1 [file Table_1.docx]

**Table S1.** The values assigned ​​to HDSS-related variables in this study.

| **HDSS-related variables** | **Assigned values** |
| --- | --- |
| **Age (years)** | |
| 65-70 | 0 |
| 71-76 | 1 |
| ＞76 | 2 |
| **Race** | |
| Black | 1 |
| White | 2 |
| Other | 3 |
| **Marital status** | |
| Single/other | 0 |
| Married | 1 |
| **Primary site** | |
| C50.0 (Nipple) | 1 |
| C50.1 (Central portion of breast) | 2 |
| C50.2 (Upper-inner quadrant of breast) | 3 |
| C50.3 (Lower-inner quadrant of breast) | 4 |
| C50.4 (Upper-outer quadrant of breast) | 5 |
| C50.5 (Lower-outer quadrant of breast) | 6 |
| C50.6 (Axillary tail of breast) | 7 |
| C50.8 (Overlapping lesion of breast) | 8 |
| C50.9 (Breast, NOS) | 9 |
| **Tumor grade** | |
| Ⅰ | 1 |
| Ⅱ | 2 |
| Ⅲ | 3 |
| Ⅳ | 4 |
| **Breast.Adjusted.AJCC.6th.Stage (Tumor stage)** | |
| Ⅰ | 1 |
| Ⅱ | 2 |
| Ⅲ | 3 |
| Ⅳ | 4 |
| **Tumor size (mm)** | |
| ＜22 | 1 |
| 22-36 | 2 |
| ＞36 | 3 |
| **Breast subtype** | |
| HR-/HER2- (Triple Negative) | 1 |
| HR-/HER2+ (HER2 enriched) | 2 |
| HR+/HER2- (Luminal A) | 3 |
| HR+/HER2+ (Luminal B) | 4 |
| **ER status** | |
| Negative | 0 |
| Positive | 1 |
| **PR status** | |
| Negative | 0 |
| Positive | 1 |
| **HER2 status** | |
| Negative | 0 |
| Positive | 1 |
| **Radiotherapy** | |
| No | 0 |
| Yes | 1 |
| **Surgery** | |
| No | 0 |
| Yes | 1 |
| **Bone metastasis** | |
| Absent | 0 |
| Present | 1 |
| **Lung metastasis** | |
| Absent | 0 |
| Present | 1 |
| **Liver metastasis** | |
| Absent | 0 |
| Present | 1 |
| **Brain metastasis** | |
| Absent | 0 |
| Present | 1 |

HDSS: heart disease specific survival
