# Supplementary material for: A novel nomogram for predicting long-term heart-disease specific survival among older female primary breast cancer patients that underwent chemotherapy: A real-world data retrospective cohort study
Source: Front Public Health. 2022 Aug 24;10:964609. doi: 10.3389/fpubh.2022.964609 (PMC9449644; doi:10.3389/fpubh.2022.964609)
Supplement: Supplementary file 2 [file Table_2.docx]

**Table S2.** The detailed scores of independent prognostic factors in the HDSS nomogram.

| **HDSS-related** independent **variables** | **Corresponding score assignments in HDSS nomogram** |
| --- | --- |
| **Age (years)** | |
| 65-70 | 45 |
| 71-76 | 54 |
| >76 | 100 |
| **Race** | |
| Black | 45 |
| White | 20 |
| Other | 0 |
| **Marital status** | |
| Single/other | 45 |
| Married | 20 |
| **Breast.Adjusted.AJCC.6th.Stage (Tumor stage)** | |
| Ⅰ | 45 |
| Ⅱ | 58 |
| Ⅲ | 84 |
| Ⅳ | 94 |
| **Surgery** | |
| No | 45 |
| Yes | 21 |
| **Radiotherapy** | |
| No | 45 |
| Yes | 17 |

HDSS: heart disease specific survival
