# Supplementary material for: A novel nomogram for predicting long-term heart-disease specific survival among older female primary breast cancer patients that underwent chemotherapy: A real-world data retrospective cohort study
Source: Front Public Health. 2022 Aug 24;10:964609. doi: 10.3389/fpubh.2022.964609 (PMC9449644; doi:10.3389/fpubh.2022.964609)
Supplement: Supplementary file 3 [file Data_Sheet_1.docx]

**Supplementary Figures**


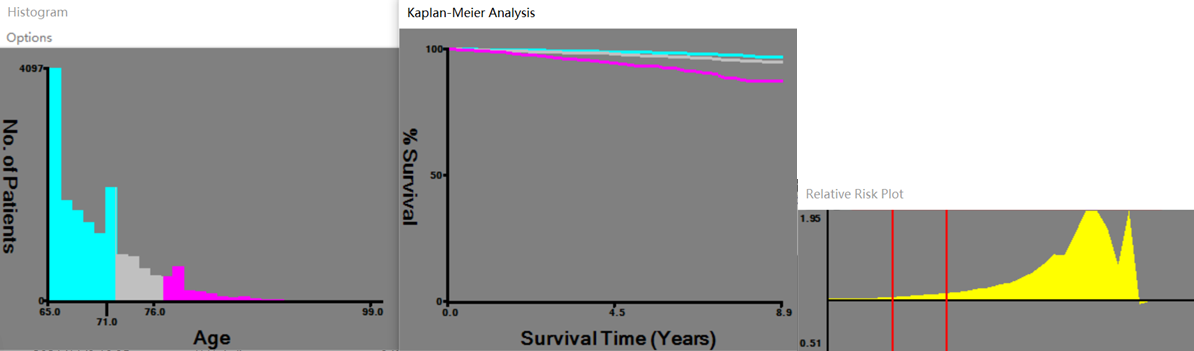


**Supplementary Figure 1:** According to the X-tile software, the optimal cut-off values for the age were determined to be 71 and 76 (years).


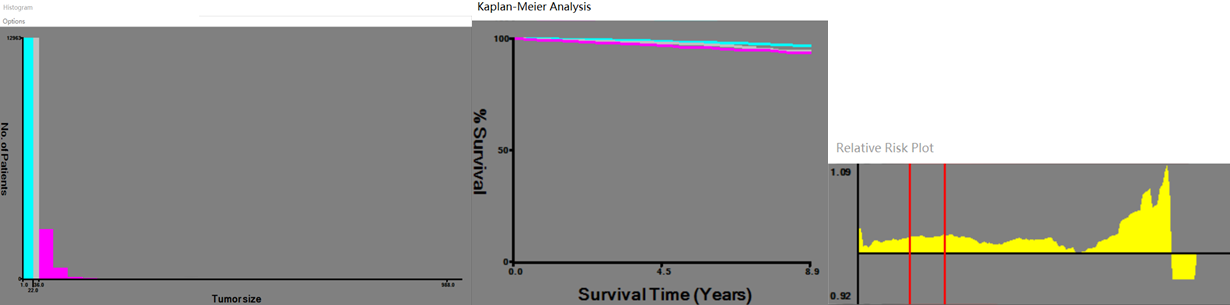


**Supplementary Figure 2:** According to the X-tile software, the optimal cut-off values for the tumor size were determined to be 22 and 36 (mm).


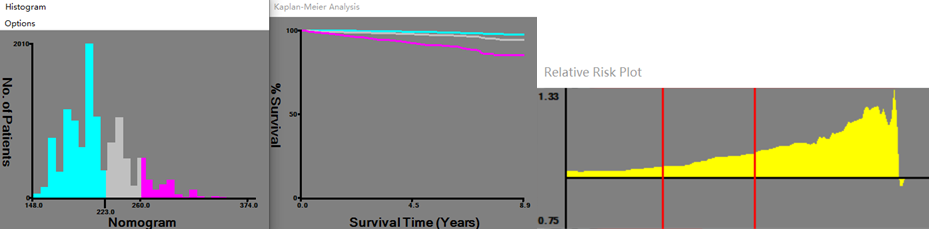


**Supplementary Figure 3:** According to the X-tile software, the optimal cut-off values for the heart-specific mortality score were determined to be 223 and 260.
